# A Clinical Review of the Treatment of Catatonia

**DOI:** 10.3389/fpsyt.2014.00181

**Published:** 2014-12-09

**Authors:** Pascal Sienaert, Dirk M. Dhossche, Davy Vancampfort, Marc De Hert, Gábor Gazdag

**Affiliations:** ^1^Department of Mood Disorders and Electroconvulsive Therapy, University Psychiatric Center, KU Leuven, Leuven, Belgium; ^2^Department of Neurosciences, KU Leuven, Leuven, Belgium; ^3^Department of Psychiatry, University of Mississippi Medical Center, Jackson, MS, USA; ^4^University Psychiatric Center, KU Leuven, Leuven, Belgium; ^5^Center for Psychiatry and Addiction Medicine, Szent István and Szent László Hospitals, Budapest, Hungary; ^6^Department of Psychiatry and Psychotherapy, Faculty of Medicine, Semmelweis University, Budapest, Hungary

**Keywords:** catatonia, benzodiazepines, electroconvulsive therapy, glutamate antagonists, zolpidem, transcranial magnetic stimulation

## Abstract

Catatonia is a severe motor syndrome with an estimated prevalence among psychiatric inpatients of about 10%. At times, it is life-threatening especially in its malignant form when complicated by fever and autonomic disturbances. Catatonia can accompany many different psychiatric illnesses and somatic diseases. In order to recognize the catatonic syndrome, apart from thorough and repeated observation, a clinical examination is needed. A screening instrument, such as the Bush-Francis Catatonia Rating Scale, can guide the clinician through the neuropsychiatric examination. Although severe and life-threatening, catatonia has a good prognosis. Research on the treatment of catatonia is scarce, but there is overwhelming clinical evidence of the efficacy of benzodiazepines, such as lorazepam, and electroconvulsive therapy.

## Introduction

Catatonia is a severe motor syndrome with an estimated prevalence among psychiatric inpatients of about 10% (1, 2). Catatonia can accompany many different psychiatric illnesses and somatic diseases. A minority of catatonic patients suffers from schizophrenia (30%), while a majority has a bipolar disorder (43%) (1, 3, 4). Catatonia has also been linked to other psychiatric disorders, such as obsessive-compulsive disorder (5), post-traumatic stress disorder (6, 7), or withdrawal from alcohol (8) or benzodiazepines (9, 10). In up to 25% of cases, catatonia is related with general medical or neurologic conditions (1, 11). Recently, it was shown repeatedly that catatonic symptoms are observable in most patients diagnosed with anti-*N*-methyl-d-aspartate receptor (anti-NMDAR) encephalitis (12, 13). In adolescents and young adults with autism, catatonia is found in 12–17% (14). Pediatric catatonia also emerges in patients with tic disorders, and a variety of other (developmental) disorders (15). The same principles of evaluation and treatment seem to apply to pediatric patients as in adult patients (15, 16).

A life-threatening situation occurs when catatonia is accompanied by fever and autonomic abnormalities. Malignant catatonia, coined as “lethal catatonia” by Stauder in 1934 (17), presents as a constellation of catatonia, stuporous exhaustion, autonomic instability, respiratory failure, collapse, coma, and often death if left untreated. This clinical picture is very close to what is observed in neuroleptic malignant syndrome (NMS), which is considered by several experts to be a drug-induced form of catatonia (18).

## Evaluation, Differential Diagnosis, and Treatment

An effective treatment starts with a swift and correct diagnosis. In any patient exhibiting marked deterioration in psychomotor function and overall responsiveness, catatonia should be considered. Moreover, any patient that is admitted to a psychiatric ward with a severe psychiatric disorder, such as depression, bipolar disorder, a psychotic disorder, or autism spectrum disorder, should be examined routinely (19). Some signs and symptoms are evident upon observation of the patient during a psychiatric interview. Other specific symptoms, however, such as automatic obedience, ambitendency, negativism should be elicited during a neuropsychiatric examination (19, 20). A rating scale can be used as a screening instrument and aid in the detection and quantification of catatonia. A number of rating scales have been found reliable, sensitive and specific: the Rogers Catatonia Scale, the Bush-Francis Catatonia Rating Scale (BFCRS) (and its revised version), the Northoff Catatonia Rating Scale, and the Braunig Catatonia Rating Scale (19). Early detection of catatonia is of great importance, since the presence of catatonic signs possesses significant prognostic and therapeutic value (19).

Unfortunately, no laboratory test specifically defines catatonia. The “diagnostic weight” of several proposed laboratory and imaging tests is limited (21). Possible laboratory tests, primarily to assess various underlying conditions, include a complete blood count and metabolic panel, erythrocyte sedimentation rate, blood urea nitrogen, creatinine, serum iron, and creatinine-phosphokinase, antinuclear antibodies, and urinalysis, and magnetic resonance imaging, electroencephalogram, cerebrospinal fluid analysis (4, 11). Given the frequent association with anti-NMDAR-encephalitis, detection of IgG antibodies to NMDAR in cerebrospinal fluid or serum is advisable (22). Since serum iron was found to be reduced in NMS compared to catatonia (23), some authors see low serum iron as a risk factor for developing NMS after using antipsychotics in a catatonic patient (24). A drug screen to detect common illicit and prescribed substances is necessary.

## Prognosis

Prognosis of catatonia is good, especially with early and aggressive treatment. In mood disorders, prognosis is probably better than in psychotic disorders. Kraepelin, who classified catatonia as a type of dementia praecox, wrote, in the 9th edition of his textbook, that almost half of catatonic attacks begin with a depressive phase and that these patients had a better prognosis. Hoch, in a monograph on benign stupors, reports good outcomes (‘*remission and return to the community*’) in 13 patients with manic-depressive illness, and a poor outcome in 12 schizophrenics (25). In more recent studies, results are conflicting. Most of the available data confirm a worse prognosis in the context of schizophrenia. In a randomized double-blind, placebo-controlled trial in 18 patients with chronic schizophrenia, who also displayed enduring catatonic features, 6 mg lorazepam per day for 12 weeks did not have any effect on catatonic symptoms (26). In another recent study, 73% of 24 patients with catatonia remitted within 6 days after starting benzodiazepines: partial responders (6/24) all had schizophrenia (27). In a retrospective study ECT was only partially efficacious in 2 of 4 patients with schizophrenic catatonia, whereas 5 out of 5 patients with mood disorder fully recovered (28). In a small open study, however, lorazepam (4–12.5 mg/day) was reported ineffective in 5 of 20 patients; all 5 with mood disorders (29). In a recent retrospective chart study on the effects of lorazepam in 107 inpatients with a primary diagnosis of catatonia, there was no significant difference between patients with mood disorder and those with a non-affective psychotic disorder (30, 31). Similarly, in an ECT-study in 22 patients, 13 (59%) of which had a mood disorder, no statistically significant difference was found in the effectiveness of the resolution of catatonic symptoms in persons with mood disorder versus schizophrenia (32), although the authors stress that in the patients with mood disorders more catatonic symptoms resolved with ECT than in the other group.

A factor that might complicate the difference in reported response rates of affective versus schizophrenic catatonia is chronicity. At least part of the studies showing a worse response in the context of schizophrenia have included patients with chronic catatonia (4, 26, 33). Some authors have suggested that “chronic catatonia” in the context of schizophrenia is phenomenologically different and less responsive to either benzodiazepines or ECT (34), and thus carries a less favorable prognosis then the acute forms of catatonia. The fact that a longer duration of the episode has been reported to predict a worse response should not be seen as a reason to withhold adequate treatment in patients presenting with longstanding or resistant catatonic symptoms. Catatonia that is not effectively treated may persist for years, but should nevertheless be promptly treated upon detection. A number of cases are illustrative of the *overall* good prognosis, even in chronic and longstanding catatonic symptoms. A man with a 17-year history of catatonia responded swiftly to the administration of lorazepam (35); a 15-year-old girl with catatonic lupus, resistant to several treatments was successfully treated with ECT after 3 months (36). A man developing catatonia after myocardial infarction remained catatonic for 1.5 years until he was treated with ECT (37). A mentally retarded boy with catatonia of 5 year’s duration improved with lorazepam (38). Both Ripley and Millson (39) and Salam et al. (40) reported positive response to lorazepam in two patients with psychogenic catatonia that lasted 2 1/2 years and 3 months, respectively. A long treatment delay should, however, be avoided since it can lead to a variety of serious medical complications, some of which may be lethal (41).

In the above mentioned retrospective lorazepam-study in 107 inpatients (30), predictors of response to lorazepam were examined. A longer illness duration, the presence of mutism, third-person auditory hallucinations and ‘made phenomena’ (in which the individual feels he is being made to do something) predicted a poor response, whereas the presence of waxy flexibility predicted a good response (30). It should be noted that low lorazepam doses (3–6 mg/day) were used. A longer illness duration also seemed to predict a worse outcome in the first 11 patients treated by László Meduna, who invented convulsive therapy in 1934 (42, 43). In a 1912 monograph, Urstein reports on 30 patients with catatonia, with various underlying conditions and a variable prognosis, being worse in patients with a higher number of episodes (44). Fink states that prognosis is especially favorable when the syndrome is dominated by stupor, hyperactivity, rapid, and pressured speech, and lability of mood, or if there is a previous episode with recovery, a rapid onset of the episode, and good social functioning prior to the episode (45, 46). Van Waarde and co-workers have examined predictors of response to ECT in 27 catatonic patients and found improvement to be significantly associated with younger age, and the presence of autonomic dysregulation, especially higher body temperature (47). There is anecdotal evidence that organic catatonia is less responsive, or does not respond at all, to ECT. Swartz and colleagues described four patients with a variety of neurological impairment (Alzheimer’s disease, post-encephalitic mental retardation, cerebellar atrophy, epilepsy, and spinal injury). Patients showed only transient and partial improvement to ECT. The authors suggest that advanced pathological changes of the CNS might explain a diminished response and that these states require a particularly intensive treatment (48).

Raffin and coworkers (16) recently reported treatments used in 66 consecutively hospitalized children and adolescents between the ages 9–19 with catatonia. The most frequent comorbid conditions were schizophrenia (*N* = 38, 58%), pervasive developmental disorder (*N* = 17, 26%), medical conditions (*N* = 16, 24%), bipolar disorder (*N* = 11, 17%), and intellectual disability (*N* = 8, 12%). The naturalistic design of the study lists a wide range of treatments in various combinations. Average catatonia scores decreased and level of function increased during the admission. Fifty-one (77%) patients received benzodiazepines, in doses up to 15 mg of lorazepam per day, which were effective in 65% of cases. Twelve (18%) received ECT, in three cases as first-line treatment (of which two cases had malignant catatonia). One patient with schizophrenia received maintenance ECT. There was no association between sociodemographic variables, except gender (males improved less than females) or co-morbid (medical or psychiatric) condition, and treatment response. Lower catatonia scores on admission and acute onset were associated with better clinical response. Cases with posturing and mannerisms were found to have less improvement than other cases.

## Treatment of Catatonia

### Supportive measures

A broad range of complications of catatonia can occur, such as aspiration pneumonia, dehydration, muscle contractures, pressure ulcers, nutritional deficiencies, severe weight loss, thiamine deficiency, electrolyte disturbances, urinary tract infections, and venous thromboembolism (11, 49, 50), some of which can lead to life-threatening situations. Some patients will require a high level of nursing care, and IV fluids and/or nasogastric tube feeds, in order to reduce the risk of morbidity and mortality caused by immobility, poor nutrition and dehydration (11). Anticoagulant therapies can be used to prevent deep vein thrombosis/pulmonary embolism in immobile patients (50). Medical complications should be treated lege artis. Given the often dramatic and prompt improvement of motor immobility after treatment, the major measure in preventing complications is a prompt diagnosis, and a rapid initiation of an adequate treatment of the catatonic state.

### Antipsychotics

All prescribed medications should be evaluated for their potential to induce catatonic symptoms and discontinued if possible. There is some ambiguity about the role of antipsychotics but it is generally encouraged to discontinue antipsychotic treatment in patients presenting with catatonia (46). In the presence of a catatonic state, both first and second generation antipsychotics (SGA) may contribute to maintaining or worsening the catatonic state and increase the risk of developing NMS (51–54). During a prospective follow-up of 82 patients that had received antipsychotics at some point when catatonic, NMS developed in three cases (3.6%) (4), a substantially higher incidence than the estimated incidence of 0.07–1.8% in all antipsychotics-treated patients (55). The risk of worsening catatonia appears greater with neuroleptics and antipsychotics with higher D2-blockade and a higher potential of causing extrapyramidal side effects (56), but a worsening of catatonia and precipitation of NMS has also been reported in association with, e.g., olanzapine (57, 58).

Although it is generally accepted that neuroleptics are ineffective in catatonia (59), the role of the SGA in the treatment of catatonia is more ambiguous, and based on cases mostly with schizophrenia (21). SGA have weak GABA-agonist activity and 5HT2-antagonism that could stimulate dopamine release in the prefrontal cortex and thus alleviate catatonic symptoms (11). Several authors have reported a beneficial effect of SGA, such as clozapine (60–63), olanzapine (64–66), risperidone (67–70), and quetiapine (71). In one randomized controlled trial, in 14 stuporous psychotic patients, risperidone (4–6 mg/day) was compared to ECT. ECT-treated patients showed significantly greater improvement than those receiving risperidone (72).

The use of antipsychotics in the presence of catatonia should be evaluated in any individual case. We support, however, the general notion to discontinue neuroleptics because of their inefficacy and their potential of aggravating the catatonic symptoms. Once treatment with benzodiazepines or ECT is started and catatonia improves, there may be a role for SGA to target residual psychotic symptoms such as delusions or hallucinations, especially in patients with schizophrenia (69), or as a prophylactic treatment in psychotic disorders and mood disorders. SGA with low D2 blockade (quetiapine, olanzapine) or with D2 partial agonism (aripiprazole) should be favored in these situations (54).

Patients presenting with both delirium and catatonia warrant special consideration (73, 74). Catatonia is a frequent feature of delirious mania, a severe syndrome characterized by the rapid onset of delirium, mania, and psychosis. Symptoms of catatonia and delirium overlap, complicating diagnosis. Moreover, DSM states that catatonia should not be diagnosed if it occurs during the course of a delirium. The issue is important because treatments for catatonia and delirium are different, albeit with overlap. While delirium is typically treated with (typical or atypical) antipsychotics, the emergence of catatonia may caution against the use of antipsychotics (75, 76). Moreover, if catatonia is not recognized in a delirious patient, the withdrawal or withholding of benzodiazepines sometimes thought to worsen delirium may induce catatonia or leave catatonia untreated. Further studies in delirious patients are needed to aid these treatment dilemmas (77).

### Diagnostic test

Benzodiazepines are the mainstay of the treatment of catatonia and are also helpful as a diagnostic probe. A positive *Lorazepam Challenge Test* validates the diagnosis of catatonia. After the patient is examined for signs of catatonia, 1 or 2 mg of lorazepam is administered intravenously. After 5 minutes, the patient is re-examined. If there has been no change, a second dose is given, and the patient is again reassessed (46, 78). A positive response is a marked reduction (e.g., at least 50%) of catatonic signs and symptoms, as measured with a standardized rating scale. Favorable responses usually occur within 10 min (46). If lorazepam is given intramuscularly or *per os*, the interval for the second dose should be longer: 15′ and 30′, respectively. Many clinicians will share the experience that a “lorazepam test” not only confirms the diagnosis of catatonia but that it also makes the underlying psychopathology apparent “*by permitting mute patients to speak*” (79). Analogous to the lorazepam test, a *Zolpidem Challenge Test* was proposed (80, 81). In this test 10 mg of zolpidem is administered *per os* and after 30 min the patient is examined. A positive response is a reduction of at least 50% of the BFCRS-score. After a positive response, treatment can be initiated.

### Benzodiazepines

Benzodiazepines are the first-choice treatment for catatonia, regardless of the underlying condition. Benzodiazepines are positive allosteric modulators of GABA-A receptors and will correct deficient GABA-ergic function in the orbitofrontal cortex (11). Following a positive *Lorazepam Challenge Test*, repeated doses of benzodiazepines can be used as a treatment. Their use is safe, easy and effective, with remission rates reported to be as high as 70–80% (4, 27, 82–87). In a naturalistic study of 66 children and adolescents with catatonia, it was found that benzodiazepines improved catatonia in 65% of cases, that there was no relation between dose and level of improvement, that the dose was higher in some cases (up to 15 mg of lorazepam) than the dose recommended in pediatric patients, and that side effects were few (16). In a recent trial in 107 adult inpatients (49% with a psychotic disorder; 44% with a mood disorder), lower success rates were reported: two thirds responded but only one third of patients remitted (30). The authors argue that the lower remission rate could be explained by a delay between illness onset and treatment (30) but the doses used in the trial (3–6 mg per day) were inadequately low. As described above, it was shown repeatedly that chronic catatonia associated with schizophrenia is less responsive to benzodiazepines. Beckmann and colleagues, in a 5-year follow-up study, found benzodiazepines ineffective in the treatment of chronic catatonic schizophrenia (33). A comparable poor response (to lorazepam 6 mg per day) was shown in a randomized double-blind, placebo-controlled trial in 18 patients with chronic catatonia in schizophrenia (26).

Efficacy of benzodiazepines in catatonia is determined by dosage (75), and doses from 8 to 24 mg lorazepam per day are common and are tolerated without ensuing sedation, especially when instituted using daily incremental dosages (77). Most authors suggest starting at 1–2 mg of lorazepam every 4–12 h, and adjusting the dose in order to relieve catatonia without sedating the patient (11). With an adequate dose, response is usually seen within 3–7 days (75), but is some cases, response can be gradual and slow (38). If high dosages of lorazepam are used, patients should be monitored carefully for excessive sedation and respiratory compromise (77). The issue of whether some benzodiazepines are more efficacious in catatonia has not been cleared. Lorazepam is generally accepted to be a first-choice drug, demonstrating a 79% remission rate and the highest frequency of use (84). Successful use of diazepam (86–90), oxazepam (91), or clonazepam (27, 92–95) has also been reported. There is no consensus on how long benzodiazepines are to be continued, and generally they are discontinued once the underlying illness has remitted. In a number of cases, however, catatonic symptoms will emerge each time lorazepam is tapered off, urging the clinician to continue benzodiazepines for an extended period of time (96, 97).

### Zolpidem

Zolpidem, a positive allosteric modulator of GABA-A receptors, seems to be a safe and effective treatment alternative. To our knowledge, Mastain and colleagues were the first to report a dramatic durable improvement of catatonia, resistant to ECT and benzodiazepines, with zolpidem in a 56-year-old woman that was in a catatonic state secondary to a subcortical stroke (98). Two years later, the same group presented an open study with zolpidem in seven catatonic patients, observing remission of catatonic symptoms in five of them within 15–30 min after ingestion, lasting 2–5 h (99). They observed these therapeutic effects at a plasma concentration between 80 and 130 ng/L. They also published a case report about catatonia in a 21-year-old woman, resolving 15 min after administration of zolpidem as the plasma concentration reached a peak level of 90 ng/mL. Relapse occurred after 4 h when plasma concentrations fell below 90 ng/mL (80). In a subsequent publication of the same French group, the authors confirm a prompt response (i.e., reduction of at least 50% of symptoms) in all patients 20 min after the administration of 10 mg zolpidem, at a plasma level of 80–150 ng/L (29). These favorable results are replicated in a few case reports (100–102). In some instances, the beneficial response to zolpidem occurred after treatment with benzodiazepines and/or ECT had failed (98, 101, 102). These data have led to the proposition of a *Zolpidem Challenge Test* (see higher), and have urged some clinicians to continue treatment with zolpidem instead of benzodiazepines, using doses from 7.5 to 40 mg per day, without noticeable adverse effects. Even though the short-half life results in a transient effect on symptoms, long-term treatment with zolpidem has also been described (101, 102).

### Glutamate antagonists

Because of its *N*-methyl-d-aspartic acid (NMDA) antagonist properties, amantadine (100–500 mg three times a day), and its derivative memantine (5–20 mg/day), have been tried in catatonia. Carroll and coworkers identified 25 cases of amantadine and memantine use in the treatment of catatonia (103). All cases (16/25 were psychotic disorders) were substantially improved, mostly after 1–7 days. It should be noted, however, that six of these cases were unpublished, and that seven other were cases experiencing a “*catatonia-parkinsonian syndrome*” while under treatment with the high-potency neuroleptic drugs haloperidol or fluphenazine. The symptoms diminished when neuroleptics were tapered and amantadine was added (104, 105). Since then, eleven additional cases describing the successful use of amantadine or memantine in catatonia have been published (58, 105–110). In one case, in an adolescent girl, catatonia that was resistant to ECT improved after the addition of amantadine (58). Only in a review of Hawkins and coworkers, a case is reported in which the use of amantadine remained without effect (84). It should be acknowledged, however, that negative cases are less likely to be published. Nevertheless, given these positive signals in the published literature, and evidence of its efficacy in treating the negative and cognitive symptoms of schizophrenia, amantadine should be further studied as a possible treatment option for catatonia.

### Other agents

There is anecdotal evidence from case-reports on the use of various other pharmacological agents, such as bromocriptine (111) and biperiden (112). Based on the GABA-hypothesis of catatonia, and the GABA-related working mechanism of several anti-convulsive mood stabilizers, these drugs have been proposed as a possible treatment option for the treatment of catatonia in bipolar patients. Only a few case-reports have been published. Valproate was used in several case reports (113–115), and found not only to have prophylactic effects but also “an ameliorating effect on the catatonic symptoms” (116). In a single case report, levetiracetam was advocated as a treatment for catatonia in bipolar disorder (117), given its possible mood stabilizing efficacy. It is of note, however, that levetiracetam has also been described to provoke catatonia (118). The use of topiramate (119) and carbamazepine (120) has also been reported. Although lithium has been anecdotally reported to have a beneficial effect on acute catatonic symptoms (121, 122), it is mostly described to be of use in the prevention of recurrent catatonia (121, 123–127), albeit with sometimes limited results (123).

### Electroconvulsive therapy

Electroconvulsive therapy should be started in a patient with catatonia that is not responding to benzodiazepines or when a decisive and rapid response is required in severe cases with life-threatening conditions such as malignant catatonia featuring high idiopathic fevers, tachycardia, severe blood pressure changes. If the underlying condition, e.g., psychotic depression, warrants ECT, this treatment may as well become the treatment of first choice.

The excellent efficacy of ECT in catatonia is generally acknowledged, even in the absence of randomized controlled evidence. The guidelines for the use of neurostimulation therapies in major depressive disorder of the Canadian Network for Mood and Anxiety Treatments define catatonia as one of the indications in which ECT should be considered as a first-line treatment (128).

Response rates in ECT are not systematically studied, but it is efficacy is described in hundreds of case reports and some small studies. In a review paper, Hawkins et al. reported an 85% (47/55) complete response rate (84). To our knowledge, only one randomized controlled ECT trial has been published. In this trial, the efficacy of ECT (BT, 3/W, *N* = 8) plus oral placebo was compared with sham ECT plus risperidone in lorazepam non-responsive non-affective catatonia. BFCRS scores reduced markedly, but the reduction was significantly more profound in the ECT group (72). Suzuki and co-workers studied both short- and long-term efficacy of ECT in intractable catatonic schizophrenia. In the acute phase, 100% of 11 patients responded to ECT. Relapse occurred in seven cases, and all occurred within 6 months. The 1-year recurrence rate was 63.6%, despite continuation pharmacotherapy (129). Relapsers received a second course of ECT followed by maintenance ECT for 1 year and four remained in remission (130). Those who relapsed again could be treated successfully with adjusting the frequency of treatment sessions (131). In another Japanese study, the efficacy of ECT was shown very clearly (132). Fifty patients presenting with catatonic symptoms, 23 of whom were diagnosed with schizophrenia, received either ECT or a benzodiazepine as first-line treatment. If benzodiazepines were ineffective, the next step was either ECT or an antipsychotic drug; if the latter failed, ECT was the last resort. Only 1 of 41 patients responded fully to benzodiazepines, and 19 responded partially. In contrast, all 17 patients who received ECT achieved remission. Payee and coworkers confirmed these favorable results: 8 of 9 (89%) lorazepam-non-responders responded to ECT (85). In another small (N = 9) prospective comparative study Escobar and colleagues (133) noted that catatonic symptoms remitted faster and to a greater extent in the depressed patients (4/9) than in those with schizophrenia (5/9).

In seven retrospective chart reviews, with a total of 222 patients, mostly benzodiazepine-non-responders, high response rates are confirmed (Table 1). The largest and most informative study included a chart review of 250 patients with catatonic schizophrenia, and was part of the Iowa 500 project (134). Eighty-five (40%) patients remitted (regardless of treatment used), while 53% of the 75 patients who had received ECT remitted. Another retrospective chart review was conducted in a university-affiliated inpatient unit. Seven of 19 patients presenting with catatonia were diagnosed with schizophrenia and ECT was used in four of them, experiencing partial (*N* = 3) or considerable (*N* = 1) improvement. In contrast, five out of five patients with mood disorder fully recovered after receiving ECT (28).

**Table 1:** **ECT in catatonia: retrospective chart reviews**.

Author(s), year	ECT EP/Schedule	Mood (%)/psychotic disorder (%)	N responders/*N* total	Responders (%)
Morrison (134)	NA/NA	0/100	40/75	53
Pataki (28)	BT/NA	56/44	6/9	67
McCall (135)	BT/NA	75/12	7/8	88
Rohland (33)	BT/3*W	59/23	26/28	93
van Waarde (47)	BT (93%)/daily [first week (56%)]	48/44	16/27	59
England (63)	BT/NA	NA	10/12	83
Raveendranathan (136)	BT/3*W	41/30	56/63	89

*EP, electrode position; BT, bitemporal; N, number; NA, not available*.

Rohland et al. reported ECT to be effective in 93% (26/28) of patients with catatonia admitted to an inpatient psychiatric unit (32). England et al. reported dramatic improvement in 10 of 12 (83%) patients with catatonia after 1–5 ECT sessions (63). In the largest study to date, 63 patients with catatonia (30% schizophrenia; 41% mood disorders) received bilateral ECT, thrice weekly, either as a first choice (*n* = 6), or after lorazepam had failed (*n* = 57). Fifty-six patients (89%) responded to ECT. Patients who responded in 4 sessions (31/56; 55%) had a lower duration of catatonia, a higher BFCRS-score, more often waxy flexibility and Gegenhalten, the involuntary resistance to passive movement of the extremities. Echophenomena predicted a slower response (136). The lowest response rates were reported in a retrospective study of 27 patients, treated with bitemporal ECT, often daily during the first week (47). Response rates were 59%. Probably, the smaller proportion of patients with a primary mood disorder, a significant treatment delay (a mean time interval of 2 months) may have negatively influenced treatment response. Another possible explanation is the fact that one third of the patients had been exposed to antipsychotics before ECT, which was reported earlier to be related to a decreased effectiveness (84).

The successful use of ECT in chronic catatonia has been described in a few case reports (137–140). Duration of catatonia varied from 3 months to 12 years, and in some cases protracted courses of bitemporal ECT (e.g., 17–68 treatments (138, 140) were needed to achieve a response. In several of these chronic cases, the catatonic symptoms reappeared when ECT was stopped. Some patients with chronic catatonia in schizophrenia will respond to a combination of ECT and clozapine. In a retrospective study, this combination resulted in sound clinical improvement as measured with the clinical global impression (CGI) in 22 patients (141).

A recent systematic review of treatment of (severe) autistic catatonia identified 12 cases with autism and catatonic symptoms of a few months to around 6 years’ duration, treated with ECT-courses that ranged from 7 to 29 sessions. Almost all cases reported a dramatic improvement with ECT, usually after relatively few sessions. A few papers report a more mixed response to ECT. Several cases reported rapid recurrence of symptoms when ECT was discontinued or suspended (142). Consoli et al collected 59 cases of children and adolescents with catatonia treated with ECT. Response to ECT was favorable for 45 patients (76%), with partial improvement noted in 3 (5%) and a lack of response in only one (143).

Recently, several authors report on the successful use of unilateral ECT in a total number of 21 cases (36, 144), 15 of which were treated with an ultra-brief pulse (UB) width (139, 145, 146). In a case-series of 5 patients with catatonia, resistant to benzodiazepines, 4 patients experienced a full response with unilateral ECT; one patient achieved only partial response, and was switched to bitemporal without experiencing any additional benefit (144). In the largest case-series to date, of 13 catatonic patients treated, 11 had rapid symptom resolution with UB right-unilateral (RUL) ECT with minimal adverse effects. Two patients who did not improve with RUL ECT were switched to bilateral ECT, which provided no additional symptom benefit (146). These favorable results probably reflect the high responsiveness of catatonia to ECT, whatever technique is used. Most authors, however, strongly recommend bilateral ECT at substantially supra-threshold stimulus dosing in severely ill patients, arguing that there is substantially more evidence, and that transient cognitive impairment is a secondary consideration in patients with severe catatonia (41, 147).

It is generally advised to stop psychopharmacological agents prior to initiation of ECT. When there was a partial response with benzodiazepine treatment, it might be unwise to abruptly discontinue this treatment, because of possible interference with seizure threshold and the risk of aggravating the catatonic state. Lorazepam and ECT can be given concurrently. If lorazepam interferes with eliciting seizures, flumazenil, a partial benzodiazepine antagonist, can be given just before the anesthetic (75).

The number of treatments, before substantial and sustained improvement becomes obvious, cannot be predicted. Often, a rapid response is seen, after one or a few treatment sessions (63, 136), but sometimes catatonia seems to require more treatments than is necessary for the relief of major depression (138, 140). Therefore, ECT treatment must be individually tailored. In severe or malignant catatonia, daily (“en bloc”) treatments for three to 5 days may be necessary. Maintenance-ECT may be useful for sustained symptom-remission.

### Transcranial magnetic stimulation

The successful use of rTMS was reported in four cases, after 7–10 high-frequency stimulation sessions of the dorso-lateral prefrontal cortex (148–151). In one case, a 45-year-old man with schizophrenia, previously recovering from catatonia with ECT failed to improve with rTMS (152).

## Conclusion

Catatonia is a severe psychomotor syndrome with an excellent prognosis if recognized and treated appropriately. The treatment of catatonia in children and adolescents should follow the same principles as in adults. Great care should be taken to avoid (medical) complications. Although a number of pharmacological agents have been tried successfully in catatonia, rarely, if ever, the effect is as immediate and dramatic as seen with benzodiazepines. If lorazepam is not available, zolpidem can be used as a diagnostic probe, and probably as a treatment alternative. If benzodiazepines fail (inadequate or transient response, excessive sedation), ECT should be started without delay. If the underlying condition warrants ECT-treatment, or in life-threatening situations like malignant catatonia or NMS, ECT is the treatment of first choice. It is advised to choose the most efficacious technique, i.e., bilateral standard-pulse ECT with a stimulus dose that is substantially above the seizure threshold.

## Conflict of Interest Statement

The authors declare that the research was conducted in the absence of any commercial or financial relationships that could be construed as a potential conflict of interest.
